# Supplementary material for: Chronic Exposure to Nitric Oxide Induces P53 Mutations and Malignant-like Features in Human Breast Epithelial Cells
Source: Biomolecules. 2023 Feb 7;13(2):311. doi: 10.3390/biom13020311 (PMC9953427; doi:10.3390/biom13020311)
Supplement: Supplementary file 1 [file biomolecules-13-00311-s001.zip › biomolecules-2176374-supplementary.pdf]

Supplemental Table S1. PCR TOPO cloning Primer Pairs Sequences and Amplicon Sizes.

Supplemental Table 1

PCR TOPO Cloning Primer Pair Sequences and Amplicon Sizes

Gene	Sequence	Exon	Amplicon Size
KRAS	Forward-GTGAGTTTGTATTAAAAGGTACTGG	2	265 bp
	Reverse-GGTCCTGCACCAGTAATATGC		
KRAS	Forward-CCAGACTGTGTTTCTCCCTTC	3	286 bp
	Reverse-TGCATGGCATTAGCAAAGAC		
PIK3CA	Forward-CTGTGAATCCAGAGGGGAAA	9	269 bp
	Reverse-ACATGCTGAGATCAGCCAAA		
PIK3CA	Forward-CATTTGCTCCAAACTGACCA	20	389 bp
	Reverse-GGTCTTTGCCTGCTGAGAGT		
TP53	Forward-CACTTGTGCCCTGACTTTCA	5	267 bp
	Reverse-AACCAGCCCTGTCGTCTCT		
TP53	Forward-CTGCTCAGATAGCGATGGTG	6	251 bp
	Reverse-CTTAACCCCTCCTCCCAGAG		
TP53	Forward-CTTGGGCCTGTGTTATCTCC	7	199 bp
	Reverse-GGGTCAGAGGCAAGCAGA		
TP53	Forward-GGGAGTAGATGGAGCCTGGT	8	274 bp
	Reverse-TAACTGCACCCTTGGTCTCC		

Supplemental Table S2. Quantitative Real-Time PCR Primer Pairs. Amplicon sizes are within the range from 80 to 250 bp.

Supplemental Table 2
Quantitative Real-Time PCR Primer Pairs

Gene	Sequence
ALDH1A1	CTCAAGGCCCTCAGATTGAC
	GTTTGGCCCCTTCTTTCTTC
OCT4	AGTGAGAGGCAACCTGGAGA
	ACACTCGGACCACATCCTTC
CD44	GGCGCAGATCGATTTGAATA
	GAAAGCCTTGCAGAGGTCAG
CD133	AATTCACCAGCAACGAGTCC
	TCCAACAATCCATTCCCTGT
CD117	AAGTGGATGGCACCTGAAAG
	AGGGGCTGCTTCCTAAAGAG
CXCR4	TTGTGCCCTTAGCCCACTAC
	CACTTCCAATTCAGCAAGCA
NANOG	CAGAAGGCCTCAGCACCTAC
	ACTGGATGTTCTGGGTCTGG
RPL18	GGATGATCCGGAAGATGAAG
	CCGCACATCATCAGTTATGG

Supplemental Table S3. Summary of Identified Mutations.

Supplemental Table 3

Summary of Identified Mutations

p53		Exon 5						Exon 6		Exon 7				Exon 8	
**Control**	CDS<br>Codon									703<br>c.703A>C<br>AAC>CAC<br>N235H<br>Substitution					
**100 mM**	CDS<br>Codon	384<br>c.384T>C<br>CCT>CCC<br>P128P<br>Transition	387<br>c.387C>T<br>GCC>GCT<br>A129A<br>Transition	386<br>c.386C>T<br>GCC>GTC<br>A129V<br>Transition	470<br>c.470T>G<br>GTC>GGC<br>V157G<br>Transversion			590<br>c.590T>C<br>GTG>GCG<br>V197A<br>Transition							
**300 mM**	CDS<br>Codon	384<br>c.384T>C<br>CCT>CCC<br>P128P<br>Transition	388<br>c.388C>A<br>CTC>ATC<br>L130I<br>Transversion	427<br>c.427G>C<br>GTG>CTG<br>V143L<br>Transversion	436<br>c.436T>C<br>TGG>CGG<br>W146R<br>Transition	488<br>c.488A>G<br>TAC>TGC<br>Y163C<br>Transition	537<br>c.537T>C<br>CAT>CAC<br>H179H<br>Transition	658<br>c.658T>G<br>TAT>GAT<br>Y220D<br>Transversion		703<br>c.703A>C<br>AAC>CAC<br>N235H<br>Substitution					
**500 mM**	CDS<br>Codon	384<br>c.384T>C<br>CCT>CCC<br>P128P<br>Transition	386<br>c.386C>T<br>GCC>GTC<br>A129V<br>Transition					658<br>c.658T>G<br>TAT>GAT<br>Y220D<br>Transversion	688<br>c.688A>T<br>ACC>AGC<br>T230S<br>Substitution	703<br>c.703A>C<br>AAC>CAC<br>N235H<br>Substitution	710<br>c.710T>A<br>ATG>AAG<br>M237K<br>Substitution	746<br>c.746G>C<br>AGG>ACG<br>R249T<br>Substitution	751<br>c.751A>C<br>ATC>ATA<br>I251I<br>Substitution	911<br>c.911C>A<br>ACC>AAC<br>T304N<br>Substitution	914<br>c.914A>G<br>AAA>AGA<br>K305R<br>Substitution

Transition	10
Transversion	5
Substitution	9

PIK3CA		Exon 9			Exon 20		
**Control**	CDS<br>Codon	1634<br>c.1634A>C<br>GAG>GCG<br>E545A<br>Transversion	1658<br>c.1658delG<br>AGT>ACC<br>S553T<br>Substitution	1659<br>c.1659T>C<br>Transition		3088<br>c.3088A>G<br>ACT>GCT<br>T1030A<br>Transition	
**100 mM**	CDS<br>Codon	1634<br>c.1634A>C<br>GAG>GCG<br>E545A<br>Transversion	1658<br>c.1658delG<br>AGT>ACC<br>S553T<br>Substitution	1659<br>c.1659T>C<br>Transition			
**300 mM**	CDS<br>Codon	1570<br>c.1570A>T<br>AGG>TGG<br>R524W<br>Transversion	1658<br>c.1658delG<br>AGT>ACC<br>S553T<br>Substitution	1659<br>c.1659T>C<br>Transition		3088<br>c.3088A>G<br>ACT>GCT<br>T1030A<br>Transition	3226<br>c.3226A>G<br>Transition
**500 mM**	CDS<br>Codon	1623<br>c.1623T>C<br>TCT>TCC<br>S541S<br>Transition	1634<br>c.1634A>C<br>GAG>GCG<br>E545A<br>Transversion	1658<br>c.1658delG<br>AGT>ACC<br>S553T<br>Substitution	1659<br>c.1659T>C<br>Transition	3224<br>c.3224A>G<br>Transition	

Transition	9
Transversion	4
Substitution	4

KRAS		Exon 1	Exon 2			
**Control**	CDS<br>Codon	89<br>c.89A>G<br>GAC>GGC<br>D30G<br>Transition	144<br>c.144A>T<br>GGA>GGT<br>G48G<br>Transversion	209<br>c.209A>G<br>CAG>CGG<br>E70R<br>Transition	234<br>c.234T>C<br>TTT>TTC<br>F78F<br>Transition	284<br>c.284A>G<br>CAT>CGT<br>H95R<br>Transition
**100 mM**	CDS<br>Codon		183<br>c.183A>G<br>CAA>CAG<br>Q61Q<br>Transition			
**300 mM**	CDS<br>Codon	86<br>c.86T>C<br>GTG>GCG<br>V29A<br>Transition	188<br>c.188A>G<br>GAG>GGG<br>E63G<br>Transition			
**500 mM**	CDS<br>Codon		162<br>c.162T>C<br>GAT>GAC<br>D54D<br>Transition	285<br>c.285T>C<br>CAT>CAC<br>H95H<br>Transition		

Transition	9
Transversion	1
Substitution	0

Supplementary Figure S1: Colony formation in SIT Media. Control and 100 µM DETANO treated cells were evaluated for growth in serum-free media supplemented with 5 ng/ml selenium, 5 g/ml insulin, and 5 g/ml transferrin (SIT). $1x10^5$ cells were plated in 60 mm dishes with SIT media and maintained for three weeks. When compared to control, MCF10A cells previously maintained in 100µM DETANO formed colonies in the SIT media.

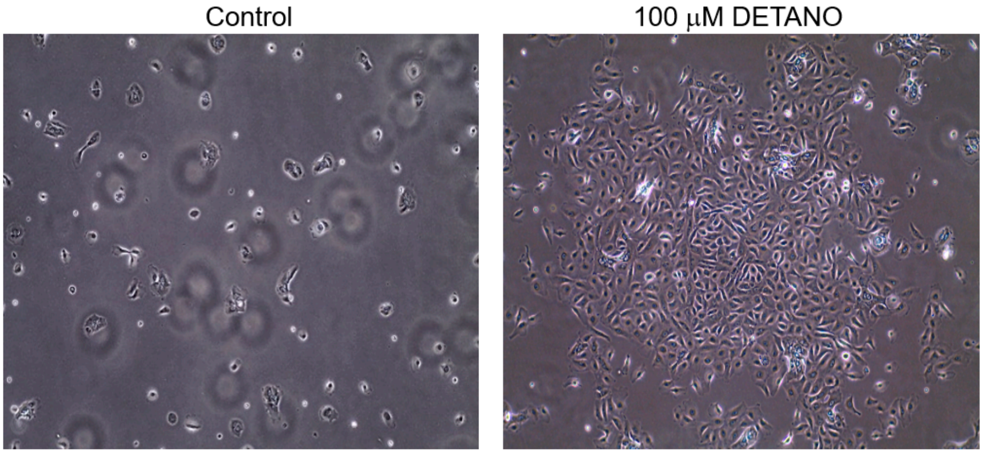

Supplementary Figure S2: Cytogenetic Analyses. Fig 4A. A dicentric Chr 1 composed by two Chr 1 long arm region 1 , band 1; a derivative Chr 1; a translocation between Chr 3 short arm region 1, band 3 and Chr 9 short arm region 2, band 2; an isochromosome 8 form by two Chr 8 long arm region 1, band 1; a translocation involved partial Chr 9 Chr 3 & Chr 5; a deletion of Chr 21; and a translocation between Chr X short arm region 2, band 2 and Chr 21 long arm region 2, band 1. Fig 4B. Chromosome 21 was showed here in green by whole chromosome painting. The Chr 21 appeared normal in this FISH assay. TIMP1 is stained in orange. An Aqua X Control probe for region Xp13.2 stain. Fig 4C. Spectrum karyotyping of Control, 100 or 1000 mM DETANO treated MCF10A cells. Control: 47, XX, del (1), +i (1q), del (3), add (8), der (9) t (5; 3; 9). 100 uM: 47, XX, dic(1;1)(q11;q11), +der(1),t(3;9)(p13;p22), der(8) i(8)(q11;q11), t(5;3;9). 1000 uM: 47, XX, dic(1;1)(q11;q11), +der(1), t(3;9)(p13;p22), i(8)(q11;q11), t(3;5;9).

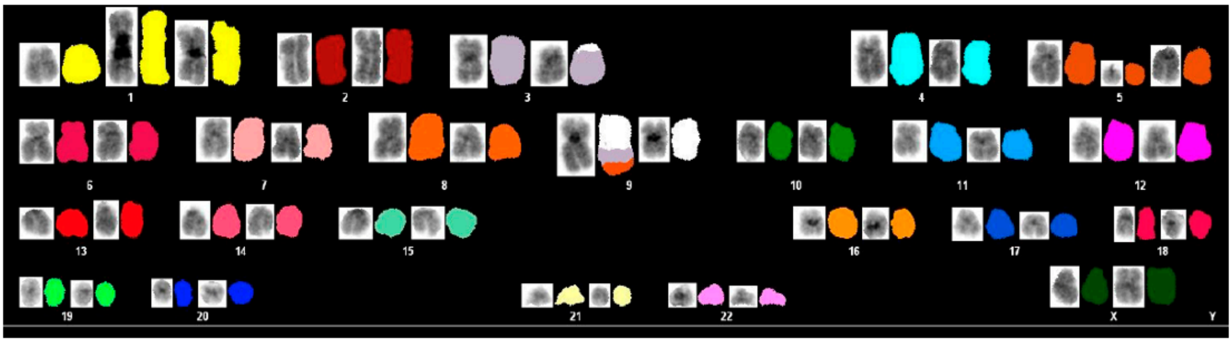

Control untreated; 47,XX, dic(1;1)(q11;q11),+der(1),-1,-3,t(3;9)(p13;22),-5,del(5),-8,i(8)(q11;q11),-9,t(9;3;5)

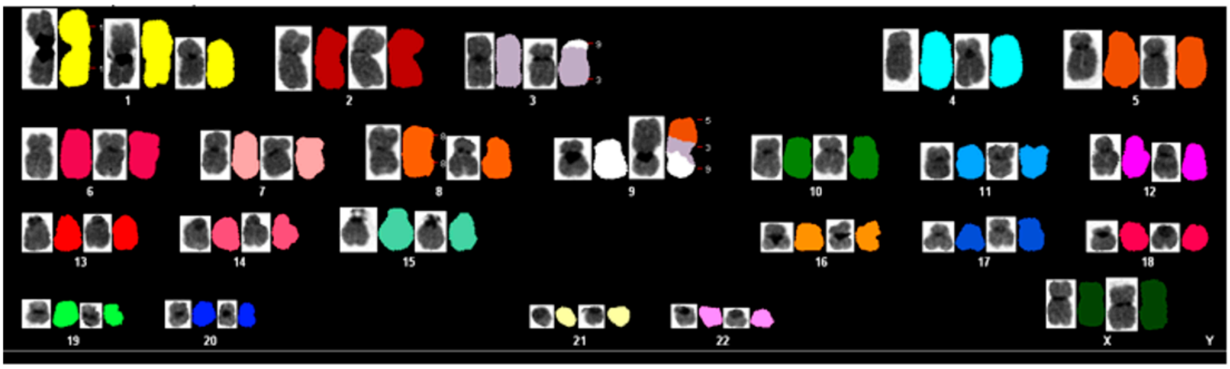

100 uM: 47, XX, dic(1;1)(q11;q11),+der(1),t(3;9)(p13;p22), der(8) i(8)(q11;q11), t(5;3;9)

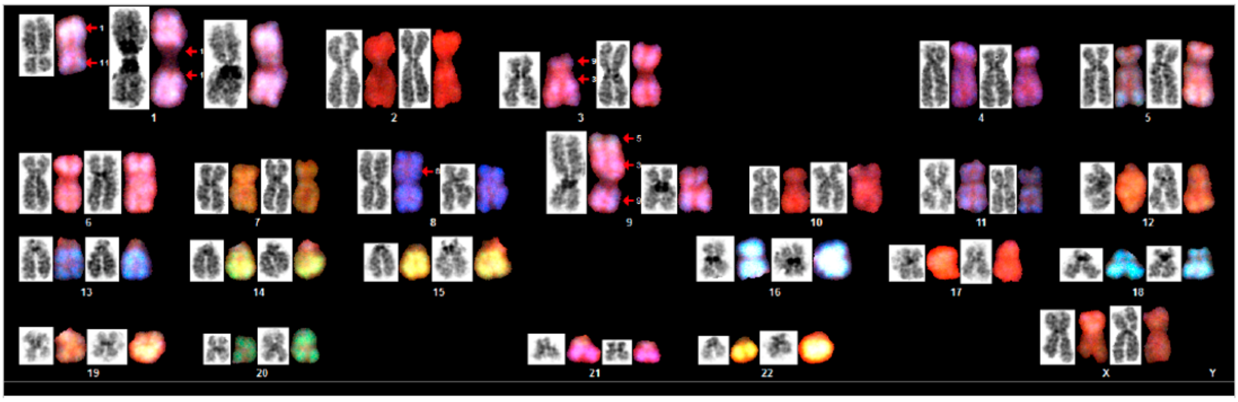

2-1 100 uM: 47,XX, der(1) t(1;11),+dic(1q;1q),der(3)t(9;3),der(8),der(9)t(9;3;5)

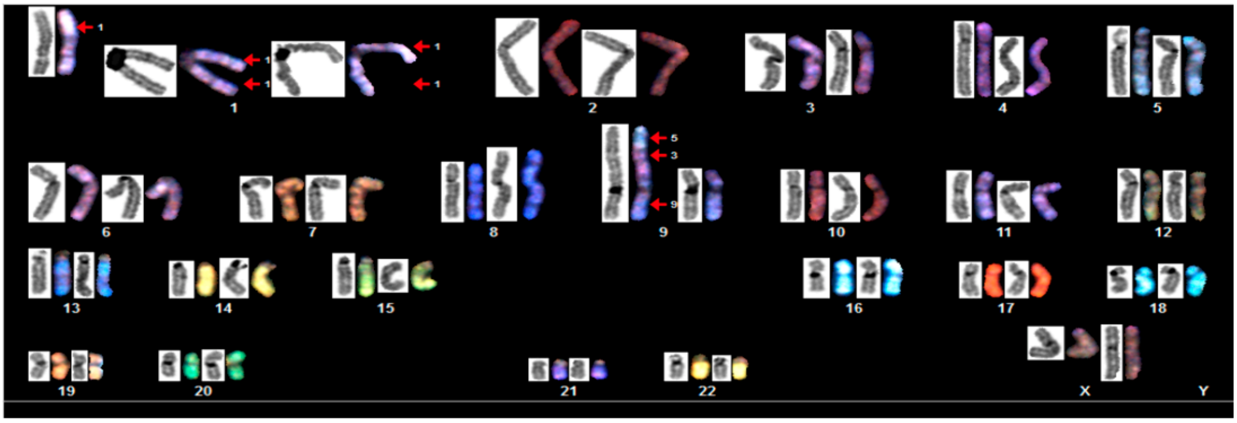

2-2 100 uM: 47,XX, der(1),+(1q), dek(3),der(8),t(9:3:5)

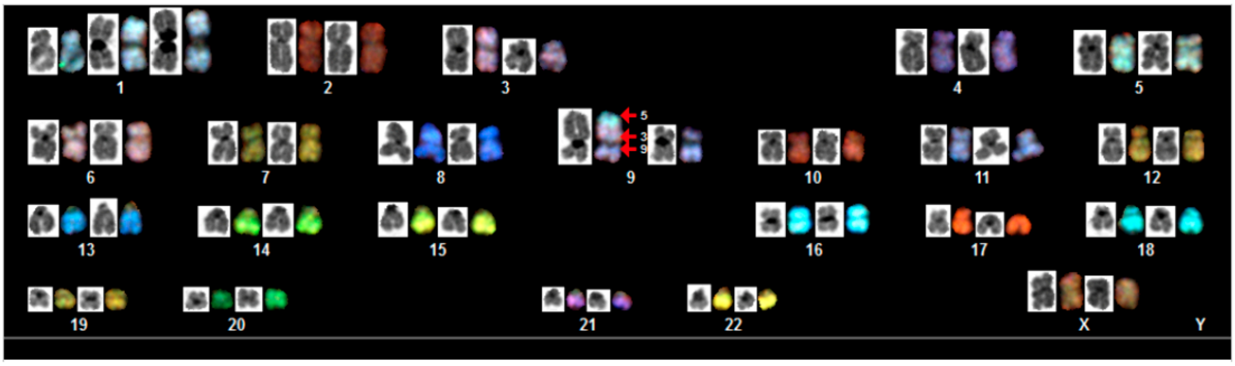

2-3 100 uM: 47,XX, der(1),+dic(1q;1q),der(3)t(9;3),der(8),der(9)t(9:3:5)
